# Integrated Photoelectrode and Electrolyte Engineering via Carbon Quantum Dots for Self‐Powered H_2_O/O_2_‐Mediated Portable Photoelectrochemical Cells

**DOI:** 10.1002/advs.76222

**Published:** 2026-06-29

**Authors:** Yang Wu, Hui‐Min Duan, Chen‐Guang Li, Tian‐Xu Zeng, Jian‐Long Li, Xue‐Tong Cheng, Yun Jing, Wei‐Zhe Li, Qing Li, Xu Tian, Jiwu Zhao, Anders Thapper, Hong‐Yan Wang

**Affiliations:** ^1^ Key Laboratory of Applied Surface and Colloid Chemistry School of Chemistry and Chemical Engineering Ministry of Education Shaanxi Normal University Xi'an China; ^2^ College of Chemical Engineering and Technology Tianshui Normal University Tianshui China; ^3^ Department of Chemistry‐Ångström Laboratory Uppsala University Uppsala Sweden

**Keywords:** carbon dots, electricity production, H_2_O/O_2_ redox‐mediators, quasi‐solid‐state photoelectrochemical cell

## Abstract

We developed N‐doped carbon quantum dots (N‐CQDs) as both photocathode modifiers and electrolytes for self‐powered portable photoelectrochemical (PEC) cells to generate electricity under visible‐light illumination. Using betaine‐type Meldonium precursor, and either ethylenediamine, *N,N*‐dimethylformamide or NH_3_·H_2_O as a nitrogen source deliver three different N‐CQDs featuring both surface‐negatively‐charged and positively‐charged groups. Due to structural‐directing template functionality of ethylenediamine, N‐CQDs(en) incorporates the highest pyridinic‐N content, which facilitates the charge conductivity and fine‐regulates the reaction selectivity within Csp^2^‐frameworks. As semiconductor‐coatings electrodeposited on Cu_2_O, N‐CQDs(en) integrate with Cu_2_O into heterojunctions (N‐CQDs(en)/Cu_2_O) to improve charge separation and promote 4e^−^ oxygen‐reduction into water. Importantly, encapsulating an aqueous solution containing N‐CQDs(en) in a gelatin/sodium L‐pyroglutamate‐derived conductor gives a quasi‐solid‐state electrolyte that facilitates the charge migration, improving the electrodes‐electrolytes interfacial incompatibility, while also possibly helping to in situ complement active sites on the modified photocathode. Coupled with a FeNiOOH/FeN‐decorated BiVO_4_ photoanode, enabling the efficient 4e^−^ water oxidation, the complete system establishes a self‐sustaining H_2_O–O_2_–H_2_O cycle. The resulting PEC cell shows impressive electricity output for over 120 h under irradiation, enough to power some small electronics. Unlike conventional photovoltaics, this cell is moisture‐tolerant, oxidation‐resistant and concurrently harnesses light and chemical energy, presenting a new paradigm for next‐generation light‐to‐electricity conversion.

## Graphical abstract

Surface‐charged N‐CQDs serve as both electrolyte additives and photocathode modifiers. Electrodeposited N‐CQDs on Cu_2_O form a stable composite, steering 4e^−^ ORR toward water. A quasi‐solid electrolyte with N‐CQDs aids charge migration, electrode‐electrolyte interfacial compatibility and replenishes active sites. Coupled with a FeNiOOH/FeN‐BiVO_4_ photoanode, the portable and self‐powered PEC‐cell operates via a regulated H_2_O─O_2_─H_2_O cycle for stable and self‐sustained electricity generation.

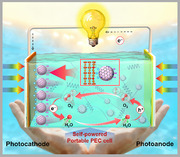

## 1 Introduction

Harnessing renewable and abundant solar energy for electricity generation has become a major research focus in recent years [1]. To date, photovoltaic (PV) cells remain the dominant technology for converting sunlight into electricity [2]. A key component of these devices is the light‐absorption layer, typically made from high‐purity monocrystalline silicon or perovskite semiconductors, which captures and transforms light into electrical energy [3]. However, these light‐absorbing materials, particularly the sensitive perovskite compounds, are highly susceptible to degradation when exposed to moisture and oxygen, significantly shortening the devices’ operational lifespan [4]. Although advanced encapsulation techniques have been developed to shield the absorbing layer from water vapor and oxygen corrosion, such protective measures substantially increase manufacturing costs. Moreover, the fundamental operating mechanism of conventional PV cells can convert light solely into electricity [3]. This inherent limitation may considerably hamper further improvements in photoelectric conversion efficiency.

By contrast, photoelectrochemical (PEC) devices leverage both light and chemical energy for power generation, which may achieve a higher output that makes them a potential breakthrough alternative to traditional PV cells [5, 6, 7]. A standard PEC cell consists of a semiconductor photoelectrode paired with a counter electrode, or a dual tandem photoelectrode system, immersed in an electrolyte solution [8, 9, 10, 11]. By combining the water oxidation reaction (WOR) at the anode with the oxygen reduction reaction (ORR) at the cathode, the clean, abundant, and eco‐friendly H_2_O/O_2_ redox mediators with the maximum four‐electron (4e^−^) migration capability can be utilized for optimized electricity power production in a PEC device under light irradiation [8, 12, 13, 14, 15]. In comparison, the dual‐photoelectrode design can generate a higher photovoltage than the single‐photoelectrode device by harvesting light on both sides, creating synergistic electric fields that suppress charge recombination [8, 16, 17]. Importantly, light irradiation on both photoelectrodes can significantly reduce the overpotentials for surface catalytic reactions, enabling the self‐powered electricity generation [8, 16, 17]. These devices function with H_2_O and O_2_ in the atmosphere, which is proposed as an important features for the next‐generation of light‐to‐electricity conversion devices.

However, electrical power generation based on H_2_O/O_2_ cycling remains challenging, primarily due to the sluggish kinetics of both the WOR and ORR, as well as the difficulty in controlling the selectivity between competing 4e^−^ and 2e^−^ transfer pathways [18, 19, 20]. More critically, dual‐photoelectrode systems necessitate meticulous optimization of interfacial compatibility and synergistic coupling between both electrodes to maximize the performance. To date, only a few studies have achieved efficient electricity generation using such configurations [8, 9, 21]. Compared to the WOR at the photoanode, the 4e^−^ ORR process to give H_2_O at the photocathode is more complex, as it involves gas–solid phase reactions and must out‐compete the 2e^−^ reduction of protons to hydrogen as well as 2e^−^ ORR for H_2_O_2_ generation [22]. Therefore, it is essential to develop robust semiconductor materials with precisely tailored 4e^−^ ORR selectivity. As a promising *p*‐type semiconductor, Cu_2_O has been developed as the photocathode material owing to its suitable bandgap for solar absorption and natural abundance [23]. Constructing heterojunctions or integrating cocatalysts with Cu_2_O can effectively mitigate its severe photo‐corrosion and rapid charge recombination, which also has the potential to promote selective reduction of O_2_ into H_2_O [24]. As a class of low‐cost zero‐dimensional carbon nanomaterials, carbon quantum dots (CQDs) are promising photocathodes modifiers for improving the PEC performance for the Cu_2_O photocathode due to their high specific surface area, tunable light‐harvesting properties, and ease of functionalization [25]. Notably, pyridinic nitrogen doping within the Csp^2^ framework of CQDs has been shown to impart pronounced 4e^−^ ORR activity [26, 27]. Unlike the commonly‐used two‐dimensional (2D) materials MXenes, which suffer from poor stability in oxygen atmospheres, or graphdiyne, which has poor aqueous dispersibility due to the hydrophobic surface, CQDs combine high oxygen stability with excellent water solubility through their hydrophilic surface functional groups. Furthermore, the small size and tunable surface charges of CQDs enable them to migrate easily in solution and function not only as electrode modifiers but also as electrolytes, a unique advantage over both 2D MXenes and graphdiyne [28, 29]. However, prolonged exposure of CQDs to corrosive electrolytes can induce severe swelling and subsequent detachment, gradually exposing the underlying material, which undermines their long‐term use in PEC systems [30]. Recent advances have demonstrated that hydrophilic hydrogels can act as effective protective overlayers that immobilize cocatalysts in a 3D porous network, thereby enhancing both PEC activity and operational stability [31]. Inspired by this, we propose that encapsulating CQDs modifiers along with an electrolyte within a hydrogel matrix will enable in situ regeneration of active sites on the photocathode, which can improve PEC device performance by maintaining structural integrity. Furthermore, the nanoporous structure of hydrogels ensures high gas permeability while retaining a sufficient water content, which is expected to further facilitate the PEC ORR process.

Besides photocatalytic selectivity regulation, the interfacial incompatibility between the electrode and electrolyte always disrupts the solid‐liquid interface junction and induces the serious photochemical‐corrosion of the electrode, leading to rapid degradation of photoelectrocatalytic performance, which is also a big challenge in PEC systems [8, 9, 10, 11]. We recently pioneered to use surface‐charged CQDs as novel electrolytes, in which the abundant surface‐anchored carboxyl (─COOH) and hydroxyl (─OH) groups enable an excellent colloidal dispersibility of CQDs in water, and the sp^2^‐hybridized carbon structure with the ordered graphitic lattices enhances the charge capacitance [21, 32, 33, 34, 35]. These endow CQDs with advantages as an efficient alternative for traditional electrolytes. We thus propose that infusing the conductive and hydrophilic hydrogels with a CQDs aqueous solution will generate a quasi‐solid‐state electrolyte. This electrolyte will act as a modifier and regenerate active sites on the photocathode in situ to significantly improve the efficiency of a PEC device, while also mitigating the leakage and ion volatilization of liquid electrolyte solutions, dramatically improving the safety of the device. Importantly, it can establish a robust ionic conduction pathway between the photoelectrode and electrolytes, improving the interfacial incompatibility. Such a “three birds with one stone” strategy will facilitate the creation of a new self‐powered, portable, and durable PEC cell.

Here, we report a self‐powered and portable PEC cell based on a dual‐photoelectrode with H_2_O/O_2_ redox‐mediators, which features nitrogen‐doped CQD (N‐CQDs), serving dual roles as both photocathode modifier and electrolyte. The device integrates a FeNiOOH/FeN‐decorated BiVO_4_ photoanode, having excellent 4e^−^ WOR capability [36], with a N‐CQDs‐electrodeposition‐modified Cu_2_O photocathode, showing a well‐regulated 4e^−^ ORR process. Identical N‐CQDs are then embedded within a pyrrolidone‐based hydrogel to form a quasi‐solid‐state electrolyte. All this together forms a PEC device, enabling a closed H_2_O‐O_2_‐H_2_O conversion cycle for the sustainable electricity generation to power LED lights and an electronic watch. The device relies on the H_2_O/O_2_ redox mediator couple, tolerating humid and oxygen containing environments, which widens the application prospect for the next generation of PEC cells.

## 2 Results and Discussion

**FIGURE 1:**
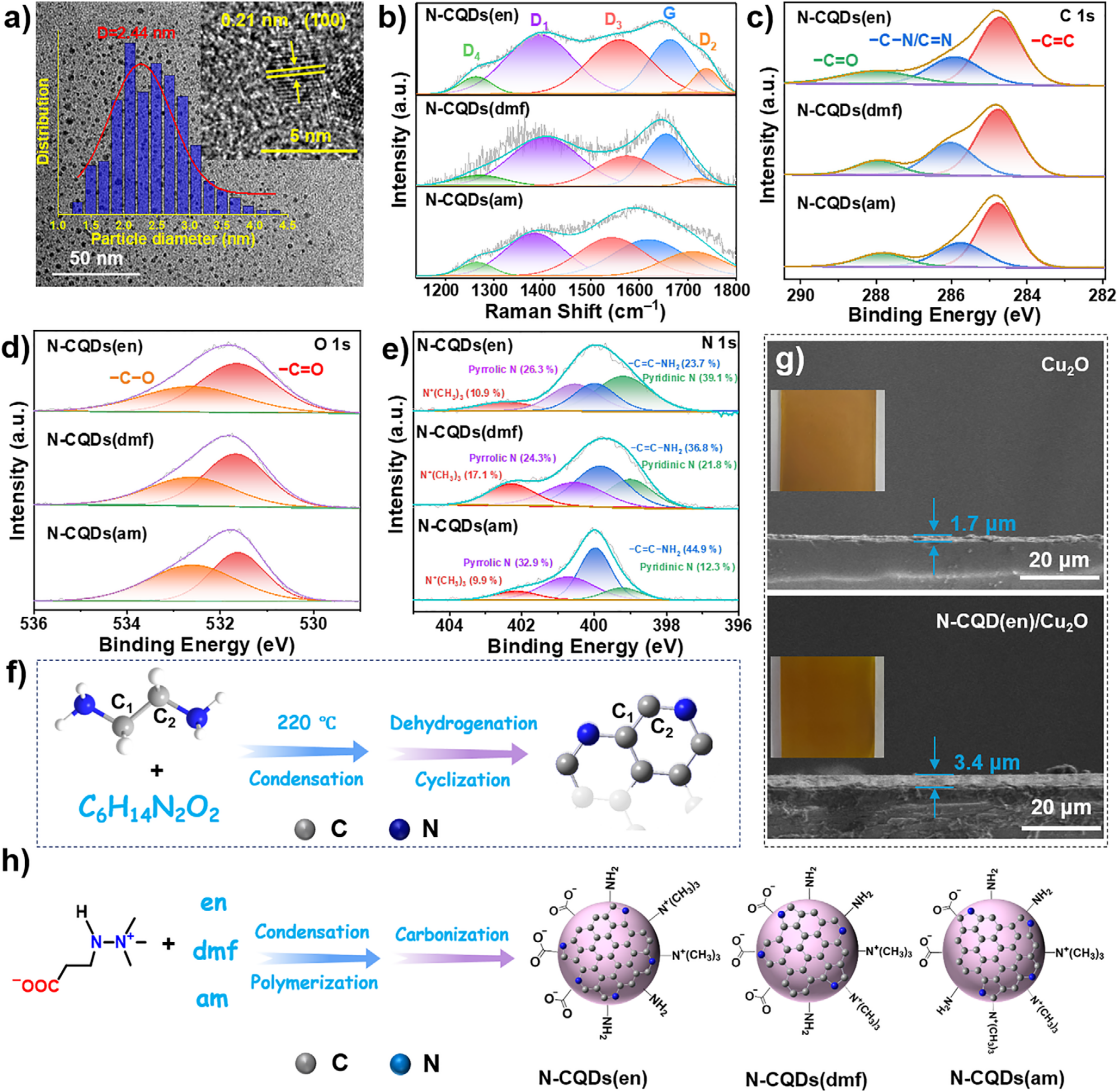
Characterization of N‐CQDs. (a) HR‐TEM of N‐CQDs(en). (b) Raman spectra and (c‐e) high‐resolution C1s, O1s, and N1s XPS spectra for the three N‐CQDs, respectively. (f) Proposed mechanism for the structural‐directing template effect of ethylenediamine. (g) SEM for the Cu_2_O and the N‐CQDs(en)/Cu_2_O photocathodes. (h) Schematic synthesis and proposed structures for the three N‐CQDs.

The N‐CQDs play a central role in the PEC cell. To eliminate the need for extrinsic inorganic ions for integrating both photoanodic part and photocathodic part as electrolytes, the N‐CQDs were designed to incorporate both positively‐charged and negatively‐charged groups on the surface. Considering the influence of pyridinic nitrogen doping within the Csp^2^ framework of CQDs on the ORR activity, three different N‐CQDs were synthesized using betaine‐type Meldonium as the precursor and either ethylenediamine, *N,N*‐dimethylformamide (DMF) or NH_3_·H_2_O as the nitrogen source via hydrothermal condensation polymerization at 220°C for 12 h. The delivered N‐CQDs were designated as N‐CQDs(en), N‐CQDs(dmf), and N‐CQDs(am), respectively. After a series of successive dialysis and freeze‐drying steps, three N‐CQDs samples were obtained as deep yellow powders, which easily transforms to a hygroscopic dark‐brown solid under ambient conditions [37]. As characterized by high‐resolution transmission electron microscopy (HR‐TEM) (Figure 1a), the N‐CQDs(en) are uniform spherical nanoparticles with a size of 2–3 nm, exhibiting a lattice spacing of 2.1 Å, which is associated with the (100) plane of graphitic domains [38].

Figure S1 exhibits the morphologies of N‐CQDs(dmf) and N‐CQDs(am) respectively, confirming their nanodot nature. Figure S2 shows the X‐Ray Diffraction spectra (XRD) for the three samples with a broad peak around 30°, corresponding to the (002) crystal plane of graphite, which suggests an overall amorphous carbon structure with short‐range order of localized graphitic assembles in the N‐CQDs [39]. Aqueous dispersion of the N‐CQDs forms transparent light‐brown solutions, with similar absorption profile in UV–vis spectroscopy (Figure S3). The spectra contain an absorption band at 300–400 nm, attributed to π→π* transitions (C═C), followed by a shoulder related to n→π* transitions for ─C═O groups, and then a tailing absorption into the visible region [40]. Aqueous dispersions of N‐CQDs exhibits excitation‐dependent fluorescence, with strong emission under 420–500 nm excitation, demonstrating efficient visible‐light harvesting and charge‐carrier generation (Figure S4) [41].

Figure 1b shows the Raman spectroscopy of three N‐CQDs samples. The D band of N‐CQDs(en) is deconvoluted into four components (D_1_–D_4_) located around 1390, 1705, 1546, and 1253 cm^−1,^ respectively, along with a G band centered at 1648 cm^−1^ [42]. It is generally accepted that the D_2_ and D_4_ bands are associated with disordered graphitic lattices, while the G band corresponds to an sp^2^‐hybridized carbon structure within an ordered graphitic framework. The D_1_ and D_3_ bands are attributed to defects in carbon rings induced by nitrogen doping into the graphitic matrix in the N‐CQDs. As shown in Figure S5, the N content in N‐CQDs(en), N‐CQDs(dmf), and N‐CQDs(am), determined from full‐scan of X‐ray photoelectron spectroscopy (XPS) spectra, is 8.12%, 7.04%, and 6.37%, respectively, indicating abundant N‐dopant within the Csp^2^ domain. Note that N‐doping can induce more catalytically active sites by the formation of electron‐rich or structural defects around the edges of CQDs. XPS fine analysis further reveals the surface functional groups and chemical composition of the three N‐CQDs samples. Typically, the high‐resolution C 1s spectrum for N‐CQDs(en) in Figure 1c is deconvoluted into four peaks: a dominant signal at 284.8 eV ascribed to C═C bonds, along with contributions at 285.9 eV associated with C─N or C═N signals, 288.1 eV assigned to C═ O bonds [43]. The presence of carboxyl groups is corroborated by the O 1s spectrum, showing signals at 531.6 and 532.6 eV in Figure 1d [44]. In combination of C 1s spectrum, this confirms the presence of ─COOH groups on N‐CQDs(en) surface. Moreover, both N‐CQDs(dmf) and N‐CQDs(am) display similar signals as N‐CQDs(en) in both the C 1s and O 1s spectra, demonstrating the existence of surface ─COOH groups. In the N 1s spectra, a peak around 402.1 eV was observed in three samples, which is assigned to protonated quaternary ammonium (N(CH_3_)_3_^+^) originated from Meldonium precursors (Figure 1e) [45]. Additionally, the N 1s signals around 399.1, 399.9, and 400.7 eV reveal the presence of pyridinic N, C═C─NH_2,_ and pyrrolic N, respectively in the N‐CQDs [46]. It demonstrates that the atomic ratio of pyridinic N in N‐CQDs(en) is 39.1%, which decreases to 21.8% in N‐CQDs(dmf) and 12.3% in N‐CQDs(am). The embedded pyridinic N within the Csp^2^ carbon framework enhances charge conductivity. Note that the nitrogen source of ethylenediamine yields the highest pyridinic N content in N‐CQDs. We propose that the two amine groups of ethylenediamine, linked by a two‐carbon chain, act as a structural‐directing template, promoting efficient intramolecular cyclization into six‐membered aromatic rings that favor pyridinic N incorporation (Figure 1f) [47]. In contrast, both ammonia and DMF rely on less efficient and more random intermolecular condensation pathways, resulting in lower selectivity for pyridinic N incorporation [48]. Note that the coexistence of positively charged N(CH_3_)_3_^+^ and negatively charged ─COO^−^ imparts a zwitterionic surface character, consistent with the observed near‐neutral zeta potential of −3.9, −2.7, and 3.3 mV for N‐CQDs(en), N‐CQDs(dmf), and N‐CQDs(am), respectively (Figure S6). Therefore, as electrolytes, all three N‐CQDs facilitate charge transport between the photoanode and photocathode. The proposed structural models of three N‐CQDs are presented in Figure 1h.

The photocathode was modified via the electrodeposition of Cu_2_O/FTO substrate into different N‐CQDs aqueous solutions with varying deposition times. A constant low bias of −0.10 V vs. Ag/AgCl, corresponding to 0.70 V versus reversible hydrogen electrode (RHE) was applied to facilitate the deposition process in the dark, as both the Cu_2_O substrate and N‐CQDs are electrochemically silent in the reaction at this potential (Figure S7). Cross‐sectional scanning electron microscopy (SEM) images confirm the successful deposition of N‐CQDs on the substrate, showing a uniform coating and a markedly greater thickness compared to the pristine Cu_2_O (Figure 1g). Based on the *J–V* characteristics of the fabricated devices, the deposition time was found to significantly influence the system's performance, with an optimal duration of 1.5 h in the dark (Figure S8). The photocathodes prepared under these conditions are denoted as N‐CQD(en)/Cu_2_O, N‐CQD(dmf)/Cu_2_O, and N‐CQD(am)/Cu_2_O, respectively.

**FIGURE 2:**
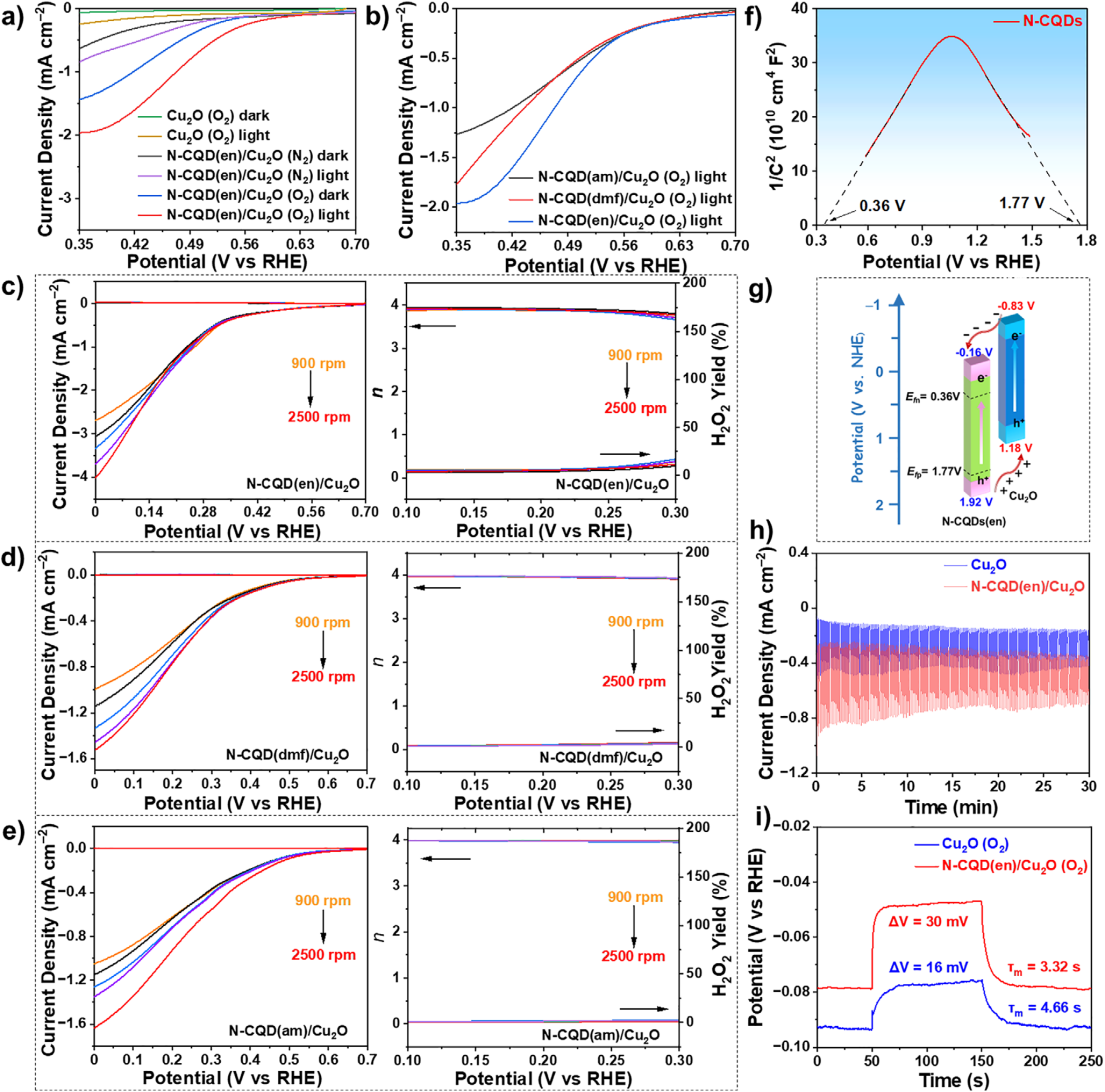
(a,b) The ORR LSV on different photocathodes (2 cm^2^) in an O_2_‐ or N_2_‐saturated buffer solution, with or without irradiation by 450 nm LED. (c–e) RRDE measurements and the corresponding *n* value and H_2_O_2_ yield on the different composite photocathodes in buffer solution. (f) Mott‐Schottky plots of N‐CQDs(en). (g) Band alignment of the N‐CQD(en)/Cu_2_O photocathode. (h) Long‐time PEC operation with chopped light irradiation, and (i) OCVD of the different photocathodes in buffer solution.

The ORR performance of the N‐CQD/Cu_2_O photocathode was evaluated in a standard three‐electrode configuration, with the photoelectrode as the working electrode, Ag/AgCl as the reference electrode, and a graphite rod as the counter electrode. The system was illuminated using commercial LEDs (λ = 450 nm, 2.60 mW·cm^−2^). Linear sweep voltammetry (LSV) was conducted in an O_2_‐saturated 0.2 m sodium carbonate‐bicarbonate buffer solution (pH = 10, conductivity = 30 mS·cm^−1^). As shown in Figure 2a,b and Figure S7, the pristine Cu_2_O photocathode exhibits negligible current response in the dark under O_2_ atmosphere, which is slightly increased under illumination. In stark contrast, the N‐CQD/Cu_2_O electrode shows a pronounced photocurrent onset, with the current increasing steadily as the potential was scanned negatively. The N‐CQD(en)/Cu_2_O photocathode exhibits the onset at approximately 0.60 V vs. RHE, reaching a current density of −1.96 mA·cm^−2^ at 0.35 V vs. RHE, as a clear indicative of ORR activity [49]. Similar PEC responses were observed when using N‐CQD(dmf)/Cu_2_O or N‐CQD(am)/Cu_2_O photocathode in buffer solution with the current density of −1.72 or −1.26 mA·cm^−2^ at 0.35 V vs. RHE, respectively (Figure S9). These results clearly demonstrate that modification with N‐CQDs significantly enhances the PEC ORR activity compared to bare Cu_2_O in O_2_‐saturated electrolyte. The photocurrent density increases with the pyridinic N content in the Csp^2^ framework, following the order of N‐CQD(en)/Cu_2_O > N‐CQD(dmf)/Cu_2_O > N‐CQD(am)/Cu_2_O. This trend implies that N‐CQDs serve as the active sites for ORR and demonstrates the role of pyridinic N in tuning ORR performance.

To evaluate the selectivity of PEC ORR on N‐CQDs modified Cu_2_O, rotating ring‐disk electrode (RRDE) measurements were conducted under irradiation from LEDs at λ = 450 nm. As shown in Figure 2c–e, the N‐CQD(en)/Cu_2_O photoelectrode exhibits a significantly enhanced disk current under illumination compared to the other electrodes, under identical experimental conditions, indicating a more efficient electron transfer pathway for ORR [50]. Usually, the PEC ORR proceeds through two parallel pathways: a direct 4e^−^ transfer producing water, and a 2e^−^ pathway yielding H_2_O_2_ as the main product. H_2_O_2_ can be oxidized at the ring electrode, so the ring current serves as an indicator of peroxide formation. Notably, the pristine Cu_2_O photoelectrode generates a substantial ring current, whereas the N‐CQD(en)/Cu_2_O photoelectrode produces negligible, suggesting minimal H_2_O_2_ formation. This implies that the N‐CQD(en) modification favors 4e^−^ reduction pathway toward water. The selectivity was further quantified by calculating the electron transfer number (*n*). As shown in Figure 2c–e, *n* remains relatively constant across various potentials. For unmodified Cu_2_O, *n* values range between 2.92 and 3.17, consistent with a mixed 2e^−^/4e^−^ process, while for N‐CQD(en) alone, *n* value increases to around 3.52 (Figure S10). In contrast, N‐CQD(en)/Cu_2_O exhibits *n* values between 3.94 and 3.98, confirming a dominant 4e^−^ reaction pathway. Figure 2d,e displays RRDE measurements for N‐CQD(dmf)/Cu_2_O and N‐CQD(am)/Cu_2_O, respectively, with both of *n* values closed to 4.0, corresponding to the 4e^−^ ORR process. Combined with the ORR performance (Figure 2b), the results show that a higher content of pyridinic N in the Csp^2^ framework enhances ORR activity but only marginally affects the selectivity. Given that carbon atoms adjacent to pyridinic N serve as primary active sites in N‐doped carbon ORR catalysts, increasing the pyridinic N content significantly improves activity [51]. The N‐CQDs(en)/Cu_2_O photocathode delivers the best performance in both 4e^−^ ORR selectivity and activity, which should give optimized electricity power production in PEC devices. Therefore, N‐CQD(en)/Cu_2_O photocathode was selected for further studies to clarify how the modifier influences the PEC performance.

To elucidate the nature of surface modifiers, XPS was employed, which provides additional insight into the surface composition of N‐CQD(en)/Cu_2_O. No significant shift in binding energy was observed between N‐CQD(en)/Cu_2_O and individual precursors (Cu_2_O and N‐CQDs), implying the interaction between Cu_2_O and N‐CQDs(en) can be attributed primarily to the electrostatic attraction during PEC operation (Figure S11) [52]. As a cathode, Cu_2_O accumulates electrons under bias, serving as the negatively charged substrate. Since the N‐CQDs surface is decorated with both negatively‐charged and positively‐charged groups, these functional groups are polarized by the external electric field, causing the separation of positive and negative charge centers [53]. It is proposed that the positively‐induced polarization acts as the driving force to trigger N‐CQDs to strongly bind and deposit at the cathode. This results in the uniform deposition of N‐CQDs onto the Cu_2_O surface.

Considering the semiconductor nature of N‐CQDs, we conducted EIS measurements to characterize the semiconductor properties of N‐CQDs(en). Figure 2f shows the capacitance values of the space charge region at various applied potentials for N‐CQDs(en). A Mott‐Schottky plot of 1/C^2^ versus potential gives two straight lines with positive and negative slopes, in different potential regimes, which correlates with n‐ and p‐type conductivities, respectively, confirming the co‐existence of n‐ and p‐type conductivities in N‐CQDs(en) [54]. Given that our previous studies indicate that the semiconductor type of N‐CQDs can be flexibly regulated by the combination of surface‐modification groups and doped heteroatoms, we assumed that the *n*‐type characteristics of N‐CQDs are mainly determined by electron transitions among the sp^2^ core and N‐dopant on the boundary [21]. In this sense, N‐CQDs(en) can provide the *n*‐type domains to interact with the p‐type Cu_2_O photocathode into a p–n junction for charge separation [55].

We further evaluated the band alignment for N‐CQD(en)/Cu_2_O. Based on the corresponding Tauc plots with (αh*v*)^2^ versus photon energy (h*v*), the band gap of N‐CQDs is determined as 2.03 eV (Figure S12). LSV in anodic and cathodic scan provides the HOMO and LUMO levels of N‐CQDs(en) estimated as 1.92 and −0.16 eV, respectively (Figure S13), giving an energy gap of 2.08 eV. This value agrees well with those obtained from optical absorption measurements in Figure S12 [56]. Extrapolating straight lines to the abscissa in the Mott‐Schottky plot gives two intercepts, representing the Fermi levels of n‐type and p‐type semiconductor, located at 0.36 and 1.77 V, respectively (Figure 2f) [54]. Characterization of band levels for Cu_2_O substrates in the buffer is shown in Figures S13–S15. Combined with the n‐type semiconductor conductivity of N‐CQDs(en), this confirms the possible formation of type II p–n heterojunctions in N‐CQD(en)/Cu_2_O (Figure 2g) [55]. Along with the band alignment of CQD(en)/Cu_2_O, N‐CQDs(en) can serve as the active site to accumulate electrons for ORR. This is in great agreement with the proposal from the activity measurements in Figure 2b, in which the ORR performance varies among the different N‐CQDs‐modified Cu_2_O photocathodes.

Moreover, a series of experiments was performed to monitor the influence of the N‐CQDs(en) modifier on the Cu_2_O photocathode. Both LSV and chronoamperometric measurements under chopped illumination reveal that the photocurrent increases proportionally with applied potential under light, while remaining near zero in the dark for N‐CQD(en)/Cu_2_O (Figure S16). The periodic and stable photo‐response confirms the high photosensitivity of the system and indicates efficient charge migration dynamics (Figure 2h). To further investigate the charge separation behavior in these systems, we extracted the photovoltage (ΔV) and employed open‐circuit voltage decay (OCVD) measurements based on the photovoltage–time profiles (Figure 2i) [57]. Under identical conditions, the N‐CQD(en)/Cu_2_O electrode exhibits a ΔV of 30 mV, compared to only 16 mV for the bare Cu_2_O. Analysis of the recombination kinetics derived from the decay yields an average charge carrier lifetime (*τ*_m_) of 3.32 s for N‐CQD/Cu_2_O, which is shorter than the 4.66 s observed for pristine Cu_2_O. This shorter lifetime indicates accelerated charge extraction and reduced recombination losses, contributing markedly to the enhanced PEC performance [57]. These findings are consistent with the EIS results, in which the N‐CQD(en)/Cu_2_O electrode displays the smaller semicircular arc compared to the pristine Cu_2_O substrate, demonstrating a substantial reduction in interfacial charge transfer resistance (Figure S17). IPCE spectra recorded under continuous illumination show higher values across all measured wavelengths for the N‐CQD(en)/Cu_2_O sample compared to the unmodified electrode (Figure S18) [58]. Extended photo‐electrolysis experiments demonstrate good operational stability, with the photocurrent remaining consistent over 14 h (Figure S19). The photocurrent difference between pristine Cu_2_O and N‐CQD(en)/Cu_2_O remains nearly constant throughout the prolonged testing, indicating a strong adhesion of the N‐CQDs layer on the Cu_2_O substrate.

**FIGURE 3:**
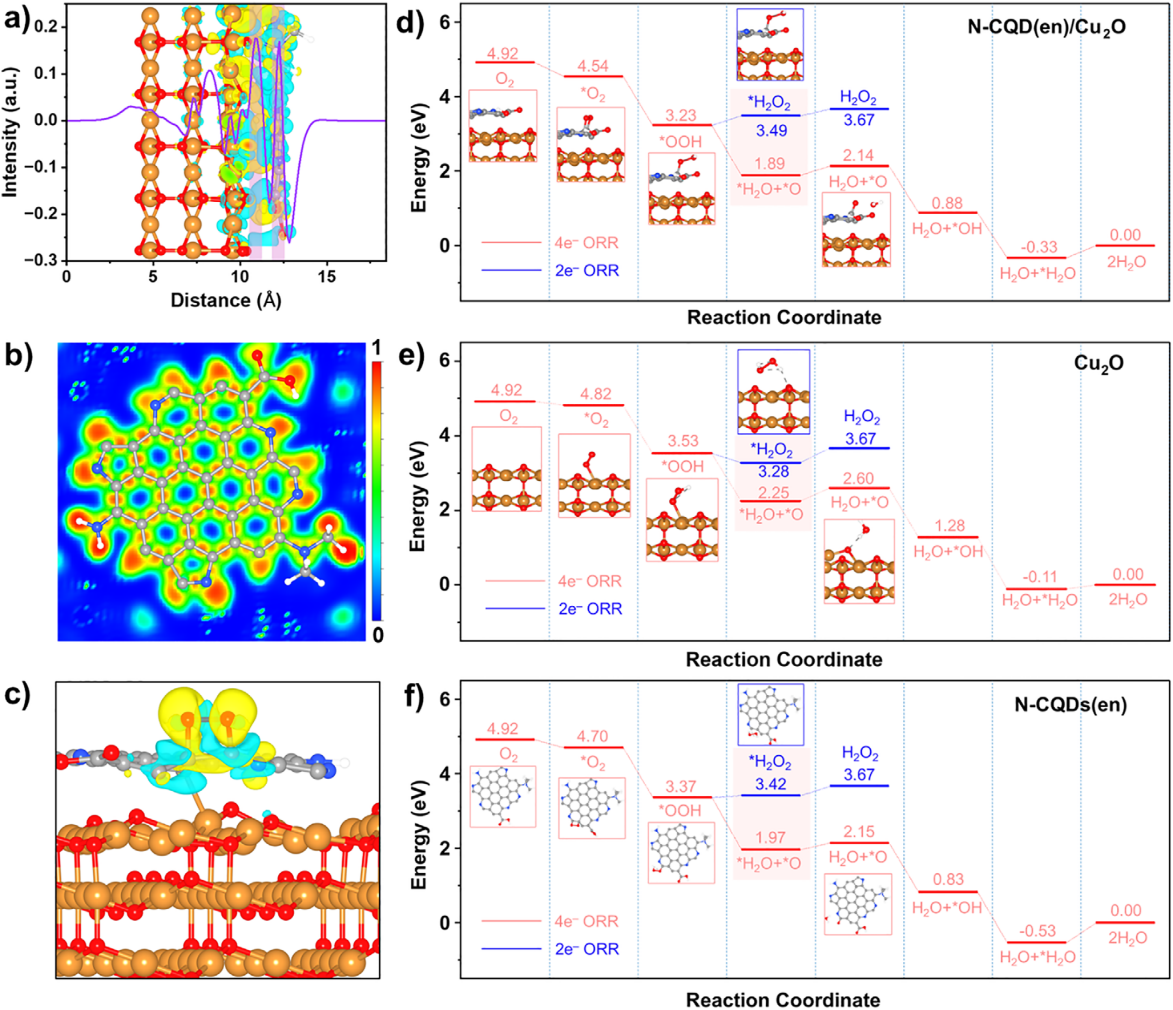
Electronic structure and Gibbs free energy. (a) Charge density difference analysis of N‐CQD(en)/Cu_2_O (The yellow and cyan bubbles represent electron accumulation and depletion regions, respectively; isovalue = 0.0005 e bohr^−3^). (b) The cross‐sectional plot of the electron ELF for N‐CQD(en)/Cu_2_O. (c) Charge density difference analysis of oxygen molecule adsorption on N‐CQD(en)/Cu_2_O (isovalue = 0.005 e bohr^−3^). Gibbs free energy profile of ORR on (d) N‐CQD(en)/Cu_2_O, (e) Cu_2_O, and (f) N‐CQDs(en), respectively.

To elucidate the heterojunction characteristics and interfacial interactions between N‐CQDs(en) and Cu_2_O, density functional theory (DFT) calculations were performed. Charge density difference (CDD) analysis reveals a strong interfacial interaction between Cu_2_O and N‐CQDs(en) (Figure 3a and Figure S20), with pronounced electron accumulation across the interface [59]. Notably, N‐CQDs(en) also exhibit significant electron enrichment, indicating efficient electron transfer from Cu_2_O to N‐CQDs(en), which establishes N‐CQDs(en) as the reduction‐active center for ORR. Furthermore, electronic localization function (ELF) analysis shows electrons are mainly localized on N‐CQDs(en) with a relatively uniform distribution (Figure 3b and Figure S21) [60]. These results collectively demonstrate that N‐CQDs(en) serve as the optimal reduction‐active site in the N‐CQD(en)/Cu_2_O heterostructure.

In comparison, pristine Cu_2_O and isolated N‐CQDs(en) exhibit limited ability to adsorb and activate O_2_ (Figure S22). In contrast, N‐CQD(en)/Cu_2_O (Figure 3c) shows markedly enhanced O_2_ activation, as evidenced by reduced electron density at the O─O bond center. The average Bader charge of 1.23 |e| indicates electron gain by the adsorbed O_2_ molecule, confirming effective reduction and activation of the O─O bond (Table S1) [61]. Correspondingly, the Gibbs free energy for O_2_ adsorption becomes exothermic (−0.37 eV, Figure 3d). Compared with Cu_2_O (Figure 3e) and N‐CQDs(en) (Figure 3f), N‐CQD(en)/Cu_2_O exhibits a stronger binding affinity toward O_2_ [62]. Moreover, the *OOH intermediate is more stably adsorbed on the heterojunction, rendering the 4e^−^ ORR pathway (*OOH → *H_2_O + *O) more competitive. In contrast, isolated N‐CQDs(en) and Cu_2_O show a competitive tendency toward both H_2_O_2_ and H_2_O formation, in good agreement with the experimental results. It is generally accepted that the 2e^−^ ORR pathway retains the peroxide bond and fails to fully release the chemical energy stored in O_2_ [61]. Therefore, the 4e^−^ ORR pathway on N‐CQD(en)/Cu_2_O is more favorable for energy conversion applications. Collectively, these results highlight the superior catalytic efficiency and effectiveness of the N‐CQD(en)/Cu_2_O heterojunction for 4e^−^ ORR pathway.

**FIGURE 4:**
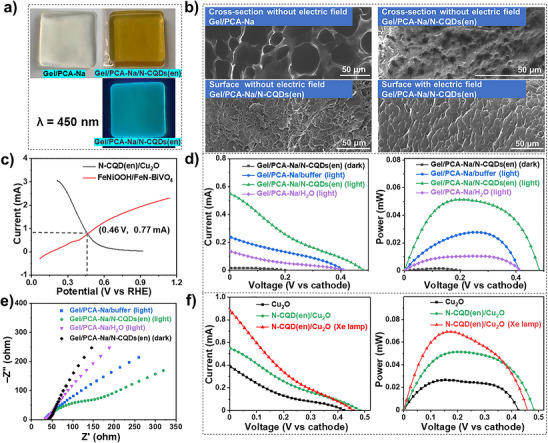
(a) Images of Gel/PCA‐Na, Gel/PCA‐Na/N‐CQDs(en), and Gel/PCA‐Na/N‐CQDs(en) under irradiation. (b) SEM images with different views of Gel/PCA‐Na and Gel/PCA‐Na/N‐CQDs(en) with or without an applied electric field. (c) LSV intersections of photoanodic and photocathodic systems in a three‐electrode configuration. (d‐e) The *J–V* curves, calculated power density curves, and EIS for N‐CQD(en)/Cu_2_O photoelectrodes in the PEC cells with different quasi‐solid‐state electrolytes under 450 nm LED irradiation. (f) The *J–V* curves and calculated power density curves for the different photoelectrodes in the PEC cells with Gel/PCA‐Na/N‐CQDs(en) electrolytes under irradiation with a 450 nm LED or a Xe lamp with a 435 nm filter.

With the aim of assembling a portable, safe, and operationally stable energy conversation device, quasi‐solid‐state electrolytes were developed. A mixture of gelatin and sodium L‐pyroglutamate (PCA‐Na) was produced, which then forms a conductive material with a transparent and jelly‐like consistency, denoted as Gel/PCA‐Na, (Figure 4a) [63]. This Gel/PCA‐Na matrix was infused with an aqueous solution of N‐CQDs to produce a quasi‐solid‐state electrolyte designated as Gel/PCA‐Na/N‐CQDs(en) (Figure 4a) [64]. To evaluate the advantages of using N‐CQDs(en) in the electrolyte, a conventional sodium carbonate–bicarbonate buffer with comparable pH and conductivity was prepared for comparison, referred to as Gel/PCA‐Na/buffer (Table S2). PCA‐Na, a key component of natural moisturizing factors, contains pyrrolidone carboxylate and sodium ions [65]. When incorporated into gelatin, it forms ionic cross‐links between amino groups in the gelatin chains and carboxylate groups of PCA‐Na, considerably enhancing the mechanical strength of the gel and promoting a more compact network structure [66]. Moreover, PCA‐Na possesses multiple hydrophilic groups that readily form hydrogen bonds with water molecules, further improving the water retention capacity of the gel. The water content was quantified using a freeze‐drying method [67]. As summarized in Table S3, Gel/PCA‐Na infused with N‐CQDs(en) maintained a water retention capacity exceeding 74%, comparable to that of pure Gel/PCA‐Na. This indicates sufficient water retention within the Gel/PCA‐Na matrix to facilitate the PEC reactions.

Figure 4b presents SEM micrographs of the Gel/PCA‐Na matrix, which exhibits an abundance of macropores, capable of accommodating N‐CQDs particles. After infusion with N‐CQDs(en), the pore size of these macropores is reduced; however, the structure remains permeable, allowing for the free migration of N‐CQDs(en) within the gel. We further examined the microstructure of Gel/PCA‐Na/N‐CQDs(en) under both the presence and absence of an external electric field. Notably, the application of an electric field results in a more ordered arrangement of Gel/PCA‐Na/N‐CQDs(en) composite. This structural ordering indicates a direct electromechanical response of the gel electrolyte based on gelatin and N‐CQDs, which means that Gel/PCA‐Na displays good charge migration ability, in contrast to the polyacrylamide (PAM) hydrogel that Wang et al. employed as a matrix of quasi‐solid‐state electrolytes for water splitting, which showed poor electroconductivity [68].

To investigate if a dual‐photoelectrode PEC cell can be assembled based on a FeNiOOH/FeN‐ decorated BiVO_4_ photoanode and a N‐CQD(en)/Cu_2_O photocathode, we recorded LSV curves for each electrode separately in a three‐electrode configuration (Figure 4c and Figure S23). The curves intersect at an open‐circuit voltage (*V*_oc_) of 0.46 V, which supports that the PEC cell should function with light energy as the only input [8, 9, 21]. The assembled dual‐photoelectrode PEC cell equipped with a highly‐water‐retaining Gel/PCA‐Na/N‐CQDs(en) quasi‐solid‐state electrolyte for an improved interfacial compatibility of the system was now examined. To maximize light absorption and power output, a photoanode and a photocathode with closely matched geometric areas were aligned under parallel illumination. Typically, the PEC cell with N‐CQD(en)/Cu_2_O photocathode and Gel/PCA‐Na/N‐CQDs(en) delivers a maximum power output (*P*_max_) of 0.052 mW, a *V*_oc_ of 0.48 V, and a short‐circuit current (*I*_sc_) of 0.56 mA, normalized to a total electrode area of 4 cm^2^, under ambient conditions with 450 nm LED irradiation (Figure 4d). In contrast, the Gel/PCA‐Na/buffer‐based device shows significantly lower performance, with a *P*_max_ of only 0.028 mW [8, 9, 21]. EIS results demonstrate that the Nyquist plots of as‐prepared cells consist of real axis intercept and semicircular opening at the high‐frequency region, representing the charge transfer resistance (*R*_ct_) and internal resistance (*R*_s_), respectively, along with a slope line at the low frequency region (Figure S24) [67]. The Gel/PCA‐Na/N‐CQD(en) electrolyte presents the smallest semicircular opening and largest slope line under irradiation, suggesting more capacitive behavior. This means that the Gel/PCA‐Na/N‐CQD(en) electrolyte possesses lower charge transfer resistance and enhanced ion transport kinetics, thereby improving the PEC performance (Figure 4e) [69]. When a bare Cu_2_O photocathode was paired with the Gel/PCA‐Na/N‐CQDs(en) electrolyte, the device achieved only a *P*_max_ of 0.027 mW, *V*_oc_ of 0.43 V, and *I*_sc_ of 0.39 mA (Figure 4f). These results confirm that the incorporation of N‐CQDs(en) to serve as both the photocathode modifier and an electrolyte, is crucial for enhancing the electricity generation performance of the PEC system. By increasing the light intensity, using an Xe lamp with a 435 nm filter, the N‐CQD(en)/Cu_2_O photocathode with Gel/PCA‐Na/N‐CQDs(en) electrolyte can generate electricity with a *V*_oc_ of 0.46 V and *I*_sc_ of 0.89 mA, along with *P*_max_ of 0.071 mW (Figure 4f). The influence of the Gel/PCA‐Na/N‐CQDs(en) electrolyte thickness on PEC cell performance was then systematically investigated. A thickness of 3 mm was found to be optimal, which is confirmed by EIS results (Figure S25).

**FIGURE 5:**
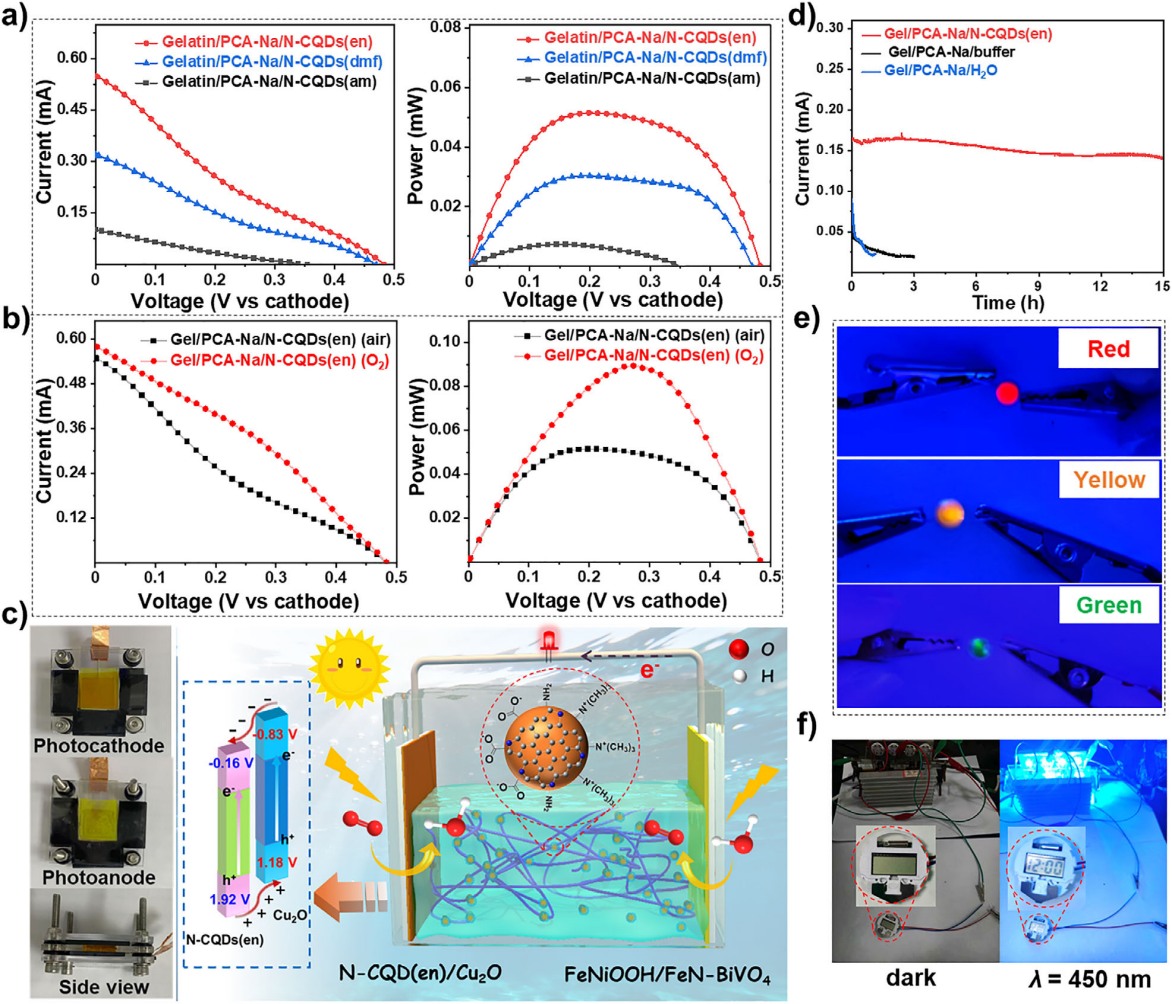
(a) The *J–V* curves and calculated power density curves for the cells with N‐CQD/Cu_2_O photocathode and the corresponding Gel/PCA‐Na/N‐CQDs electrolytes under 450 nm LED irradiation. (b) The *J–V* curves and calculated power density curves for the cell with N‐CQD(en)/Cu_2_O photocathode and the Gel/PCA‐Na/N‐CQDs(en) electrolyte under 450 nm LED irradiation in air or O_2_‐saturated conditions. (c) Different views of the PEC cell and proposed mechanism of the PEC cell for electricity generation. (d) *I–t* curves of an N‐CQD(en)/Cu_2_O photocathode with different quasi‐solid‐state electrolytes under 450 nm LED irradiation. (e) Images showing a red, yellow, and green LED powered by the light‐driven PEC cell under Xe lamp irradiation using a 435 nm filter. (f) The 3‐tandem PEC cell powering electronic watch with a power of 0.1–1.0 W.

At this point, PEC cells utilizing the three different N‐CQDs were compared. The cell with the N‐CQD(en) as photocathode modifier and quasi‐solid‐state electrolyte filler outperformed those made from N‐CQDs(dmf) and N‐CQDs(am) under the identical conditions (Figure 5a). This is in line with the 4e^−^ PEC ORR activity observed previously. In the PEC cell containing N‐CQDs(en) on the photocathode and in the electrolyte, purging O_2_ gas into the electrolyte increases the *P*_max_ to 0.091 mW, accompanied by a *V*_oc_ of 0.48 V and an *I*_sc_ of 0.58 mA under 450 nm LED illumination (Figure 5b). Under O_2_‐saturated conditions, the solar‐to‐electricity conversion efficiency (*η*) was estimated to be 0.22% after accounting for the dark current contribution, representing a 1.8‐fold enhancement compared to performance under ambient conditions. This confirms the beneficial role of dissolved O_2_ in enhancing electrical output (Table S4) [8].

Based on these findings, we propose that the electricity generation mechanism involves a H_2_O─O_2─_H_2_O cycle between the photoanode and photocathode. Upon illumination, both semiconductors generate electron–hole pairs. Photogenerated holes accumulate at the photoanode/electrolyte interface and drive the WOR, producing O_2_. The O_2_ rapidly diffuses to the cathode through the perforated channel in the semi‐solid electrolyte, increasing the local dissolved O_2_ concentration, which is reduced to water by electrons at the N‐CQD/Cu_2_O photocathode surface via 4e^−^ ORR pathway. Simultaneously, electrons from the photoanode travel through the external circuit to recombine with holes at the photocathode, producing electricity. Specifically, N‐CQDs(en) facilitate ORR activity, thereby promoting the charge transfer capacity. Assisted by the Gel/PCA‐Na/N‐CQDs(en) quasi‐solid‐state electrolyte to facilitate the mobility of N‐CQDs(en), water molecules, and oxygen between photo‐electrodes and electrolyte, this configuration utilizes the H_2_O/O_2_ couple as a redox shuttle, with a self‐sustaining H_2_O─O_2_─H_2_O cycle for stable and continuous electricity output over extended periods (Figure 5d).

Furthermore, the operational stability of the system was evaluated. As shown in Figure 5d, the optimized PEC cell delivers a highly stable photocurrent, retaining 90% of its initial value after 15 h of continuous operation. In contrast, systems employing a combination of Gel/PCA‐Na/buffer or Gel/PCA‐Na/H_2_O with N‐CQD(en)/Cu_2_O photocathode exhibit a strongly decreased performance. The use of Gel/PCA‐Na/N‐CQDs(en) effectively suppresses film detachment, giving markedly superior operational stability (Figure S26). We assume that N‐CQDs within a hydrogel matrix enable the in situ regeneration of active sites on the photocathode, which can improve the PEC device performance by maintaining structural integrity. This proposal can be further supported by the SEM of N‐CQD(en)/Cu_2_O photocathode after long‐term PEC operation, shown in Figure S27.

To illustrate the practical electricity generation capability of the device under illumination, a commercial red, yellow and green LED was powered using the PEC cell. Two tandem‐connected optimized cells were assembled as a proof‐of‐concept energy generation system. Under 450 nm LED light irradiation, this PEC device successfully illuminates the red LED with no detectable decrease in brightness over 120 h (Figure S28) [70], confirming sustained and stable light‐to‐electricity conversion. Natural sunlight could be also used for powering the LED light, but the brightness varies with the incident light intensity. When the PEC device is illuminated with a Xe lamp, the LED light is much brighter (Figure 5e and Figure S29). The proof‐of‐concept device can also provide continuous, self‐sufficient, and sustained power for an electronic watch, as shown in Figure 5f.

The performance of the device for electricity production was compared with other reported unbiased dual‐photoelectrode systems (Table S5). Clearly, our system generates electricity at much milder conditions, and its performance is above average compared with reported systems [10, 71, 72]. The cell can tolerate moisture and the presence of oxygen, which can simplify the manufacturing, improve its applicability, and ultimately make it more economically feasible for large‐scale electricity production. Finally, we designed an application scenario for our PEC cell as a light intensity meter in a greenhouse (Figure S30). The intensity of the powered LED light can be used to monitor the light conditions in the greenhouse in a continuous and real‐time manner, thereby reinforcing the interaction with users [73].

## 3 Conclusion

In conclusion, we have successfully applied novel N‐CQDs as both electrolytes and photocathode modifiers to construct a self‐driven PEC cell for electricity generation under visible‐light illumination. The N‐CQDs were designed with N‐doping into Csp^2^ domains, bearing both surface‐anchored negatively‐charged and positively‐charged groups. This allows the charged N‐CQDs to associate with both the photoanode and photocathode. By adjusting the nitrogen sources of N‐CQDs, it demonstrates that ethylenediamine displays the structural‐directing template functionality that promotes intramolecular cyclization into six‐membered aromatic rings to favor the pyridinic N‐doping. The obtained N‐CQDs(en) can be electro‐deposited on a Cu_2_O substrate and form a stable incorporated photocathode. The N‐CQDs(en) act as the active sites to promote the 4e^−^ pathway for O_2_ reduction into water on this photocathode. Encapsulating an aqueous solution containing N‐CQDs(en) in a conducting material derived from a gel and PCA‐Na, gives a quasi‐solid‐state electrolyte that enables charge migration, while also possibly helping to complement active sites on the photocathode. Coupled with a FeNiOOH/FeN‐decorated BiVO_4_ photoanode, a self‐powered, stable, and sustainable PEC cell for electricity output is produced. Since the PEC cell involves an H_2_O/O_2_ redox mediator, it can tolerate moisture and an oxygen‐containing atmosphere, which allows the device to be assembled without the need of expensive and complex packaging technology. This work thus advances practical dual‐photoelectrode cells for sustainable solar electricity generation via an efficient, low‐cost, and portable pathway, which provides valuable insights into the design of next‐generation solar cells.

## Author Contributions

**Hui‐Min Duan**: methodology, software, data curation, investigation, validation, formal analysis, visualization. **Chen‐Guang Li**: methodology, data curation, validation, investigation, software. **Jian‐Long Li**: methodology, software, data curation, investigation, validation, formal analysis. **Yang Wu**: methodology, software, data curation, investigation, validation, formal analysis, visualization, writing – original draft. **Wei‐Zhe Li**: methodology, software, data curation, investigation, validation. **Xue‐Tong Cheng**: methodology, software, data curation, validation, investigation. **Tian‐Xu Zeng**: methodology, software, data curation, investigation, validation, formal analysis. **Jiwu Zhao**: conceptualization, funding acquisition, writing – original draft, formal analysis, resources, supervision. **Anders Thapper**: conceptualization, funding acquisition, writing – review and editing, formal analysis, supervision, resources, project administration, visualization. **Xu Tian**: methodology, software, data curation, investigation. **Hong‐Yan Wang**: investigation, conceptualization, formal analysis, project administration, supervision, resources, funding acquisition, writing – original draft, writing – review and editing, visualization. **Qing Li**: methodology, software, data curation, investigation, validation. **Yun Jing**: methodology, software, data curation, investigation, validation.

## Conflicts of Interest

The authors declare no conflicts of interest.

## Supporting information




**Supporting File**: advs76222‐sup‐0001‐SuppMat.docx.

## Data Availability

The data that support the findings of this study are available from the corresponding author upon reasonable request.
