# Supplementary material for: Integrated Photoelectrode and Electrolyte Engineering via Carbon Quantum Dots for Self‐Powered H2O/O2‐Mediated Portable Photoelectrochemical Cells
Source: Adv Sci (Weinh). 2026 Jun 29;13(52):e76222. doi: 10.1002/advs.76222 (PMC13336404; doi:10.1002/advs.76222)
Supplement: Supplementary file 1 [file ADVS-13-e76222-s001.docx]

*Supporting Information*

Integrated Photoelectrode and Electrolyte Engineering via Carbon Quantum Dots for Self-Powered H_2_O/O_2_-Mediated Portable Photoelectrochemical Cells

*Yang Wu^a^, Hui-Min Duan^a^, Chen-Guang Li^a^, Tian-Xu Zeng^a^, Jian-Long Li^b^, Xue-Tong Cheng^a^, Yun Jing^a^, Wei-Zhe Li^a^, Qing Li^a^, Xu Tian^a^, Jiwu Zhao^a*^, Anders Thapper**^c*^, Hong-Yan Wang**^a*^*

^a^ Key Laboratory of Applied Surface and Colloid Chemistry, Ministry of Education, School of Chemistry and Chemical Engineering, Shaanxi Normal University, Xi’an 710119, China.

^b^ College of Chemical Engineering and Technology, Tianshui Normal University, Tianshui, 741000, China.

^c^ Department of Chemistry-Ångström Laboratory Uppsala University P.O. Box 523, 75120 Uppsala, Sweden.

Correspondence E-mail: [hongyan-wang@snnu.edu.cn](mailto:hongyan-wang@snnu.edu.cn); [anders.thapper@kemi.uu.se](mailto:anders.thapper@kemi.uu.se); [jwz@snnu.edu.cn](mailto:jwz@snnu.edu.cn)

**Table of Contents**

1. Experimental details	3
2. Figures and Tables	9
3. References	32

**1. Experimental details**

**1.1 Materials**

Betaine-type Meldonium, ethylenediamine, *N,N*-dimethylformamide (DMF) and NH_3_·H_2_O for the synthesis of N-CQDs were purchased from Energy Chemical Co. Ltd. Bismuth nitrate pentahydrate and vanadyl acetylacetonate, used for the synthesis of FeNiOOH/FeN-BiVO_4_ photoanode were obtained from Sinopharm Chemical Reagent Co. Ltd, in which the modifier precursor ferric acetate and 1,10-phenanthroline were sourced from Innochem Chemical Reagent Co., and magnesium oxide was obtained from Leyan. com. Copper (II) sulfate pentahydrate and sodium lactate used for the synthesis of Cu_2_O, were purchased from Innochem Chemical Reagent Co. and Shanghai Hushi Chemical Reagent Co., respectively. Sodium L-pyroglutamate (PCA-Na) and gelatin (from bovine skin, gel strength ~300g bloom), used for the synthesis of the Gel/PCA-Na/N-CQDs electrolyte, were purchased from Bide Pharm Chemical Reagent Co. and Energy Chemical Co. Ltd., respectively.

**1.2 Characterization**

High-resolution transmission electron microscopy (HR-TEM) images were collected on a Tecnai G2 F20 electron microscope at 200 kV. A SU8220 high-resolution field-emission scanning electron microscope was used to characterize the morphology of the materials. A Bruker D8 Advance X-ray powder diffractometer (XRD) was used to characterize the structures of materials. Raman spectra were measured on a Renishaw inVia with 532 nm laser source. X-ray photoelectron spectroscopy (XPS) analysis of the N-CQDs was carried out on a Kratos AXIS ULTRA DLD XPS instrument. The C 1s peak at 284.8 eV was chosen as the calibration for binding energy. UV-vis absorption spectra were recorded on a Hitachi U-3900 UV-vis spectrophotometer. The diffuse reflection spectrum of N-CQDs powder was also measured, and was converted from reflection to absorbance, on a Hitachi U-3900 UV-vis Spectrophotometer. The photoluminescent (PL) spectra were recorded on a Hitachi F-7000 fluorescence spectrometer. Zeta potential measurements were conducted on a Malvern Nano ZS90 particle size potentiometer. The pH of aqueous solutions was measured using an Orion star A211 pH meter from Thermo Fisher and electro-conductivity was measured on a DDSJ-319L conductivity meter.

**1.3 Preparation of N-CQDs, Electrolyte and photoelectrodes**

**1.3.1 Preparation of N-CQDs**

All the N-CQDs samples were prepared via a one-pot hydrothermal-assisted condensation polymerization based on previously reported method.^[1]^

N-CQDs were prepared with betaine-type Meldonium as the precursor, accompanied by the different nitrogen sources. Typically, betaine-type Meldonium (1.46 g, 10 mmol) was dissolved in 10 mL deionized water, followed by the addition of ethylenediamine (30 µL, 0.45 mmol), which was kept in an autoclave at 220 °C for 12 h. The resulting brown solution was filtered through a filter with pore diameter of 0.22 μm. Then, the solution was dialyzed by a cellulose dialysis membrane in 1 L deionized water under subsequent stirring. Every 4 h, fresh deionized water was applied and the whole dialysis process lasted for one day. The obtained solution was freeze-dried, which gave N-CQDs(en) as a light-yellow powder. The synthesis of N-CQDs(dmf) and N-CQDs(am) is based on the same method except that ethylenediamine was replaced by *N,*N-dimethylformamide (DMF, 35 µL, 0.45 mmol) or NH_3_·H_2_O (30 µL, 0.44 mmol), respectively. The materials are hydrophilic, and easily becomes sticky with an associated color change to dark brown once exposed to ambient conditions.^[2]^

According to the crosslinking-induced nucleation and carbonization (CINC) mechanism,^[3]^ the hydrothermal synthesis of N-CQDs proceeds via four stages: (1) condensation between the carboxyl groups of Meldonium and the amine groups of ethylenediamine, and the subsequently continuous polymerization, forms amide linkages; (2) extensive crosslinking due to the bifunctional nature of ethylenediamine, which increases the hydrophobicity of the polymer network; (3) hydrophilic/hydrophobic microphase separation, which serves as the driving force for nucleation; (4) dehydration-driven carbonization within the hydrophobic domains, transforming the sp^3^-hybridized polymer network into an sp^2^-hybridized carbon core. The proposed mechanism for the formation of N-CQDs is summarized in Figure 1h.

**1.3.2 Preparation of Quasi-Solid-State Electrolyte**

Gelatin (2 g) was dissolved into 7 mL deionized water and then the system was allowed to swell for 1 h. Subsequently, PCA-Na (1 mL, 3.9 mmol) was added and the mixture was heated in a water bath at 50 °C for 1 h.^[4]^ While still in the water bath, 5 mL different N‑CQDs or sodium carbonate–bicarbonate buffer solution at almost the identical pH and conductivity was incorporated by stirring until a homogeneous mixture was obtained (Table S2). The resulting solution was dropped into pre‑heated molds with varying thicknesses. The system was then solidified at room temperature, yielding the corresponding Gel/PCA-Na/N‑CQDs or Gel/PCA-Na/buffer composite, respectively.

**1.3.3 Preparation of the** **FeNiOOH/FeN-BiVO_4_ photoanode,** **Cu_2_O photocathode and** **N-CQD/Cu_2_O photocathode**

The preparation of the FeNiOOH/FeN-BiVO_4_ covered FTO photocathode was based on a published method.^[5]^ The preparation of Cu_2_O covered FTO photocathode followed a modified protocol based on the literature,^[6]^ and was then characterized by XRD shown in Figure S1b. Each photoelectrode was prepared using a 10 cm^2^ FTO electrode covered with 4 cm^2^ semiconductor material.

The modification of the photocathode was achieved via electrodeposition on the Cu_2_O/FTO substrate submerged in the different N-CQDs aqueous solutions with varying deposition times. In order to compare the activity of N-CQDs modified Cu_2_O photocathode, the conductivity and pH of each N-CQDs solution was adjusted to almost identical values, as shown in Table S2. The electrodeposition was carried out in a standard three-electrode configuration, with the photoelectrode as the working electrode, Ag/AgCl (with saturated KCl aqueous solution) as the reference electrode, and a graphite rod as the counter electrode. A constant low bias of -0.10 V vs. Ag/AgCl corresponding to 0.70 V versus Reversible Hydrogen Electrode (RHE) was applied under dark conditions.

**1.4 Electrochemical measurements**

**1.4.1 Photoelectrochemical (PEC) measurements for the O_2_ reduction reaction (ORR)**

The electrochemical experiments were performed on a CHI660E instrument electrochemical analyzer at ambient conditions, unless otherwise stated. The system was irradiated using a 450 nm LED lamp with the input optical power of 2.60 mW cm^−2^. Before the PEC ORR was carried out, O_2_ was continuously pumped into the buffer solution for 15 min. The photocurrent density was determined by dividing photocurrent by photoelectrodes areas. The measurement was carried out in a three-electrode configuration with the N-CQDs modified Cu_2_O/FTO photoelectrode as a working electrode, Ag/AgCl electrode (with saturated KCl aqueous solution) as a reference electrode and graphite rod as a counter electrode, in which sodium carbonate–bicarbonate buffer solution was applied as the electrolytes at pH 10.05 and conductivity of 36.20 mS·cm^−1^. The potential reported in the work are converted to RHE via calibration using the following equation: *E* (vs RHE) = *E* (vs Ag/AgCl) + 0.197 + 0.0591 × pH. Unless otherwise stated, all potentials are reported versus RHE.

**1.4.2 PEC cell**

In the PEC cell, the two-electrode system was equipped with a FeNiOOH/FeN-BiVO_4_ photoanode and a N-CQD/Cu_2_O photocathode. Gel/PCA-Na/N-CQDs or Gel/PCA-Na/buffer was employed as the quasi-solid-state electrolyte. Usually, the same N-CQDs were used to fill up the Gel/PCA-Na and modify the Cu_2_O substrate unless otherwise stated. The system was irradiated using a 450 nm LED lamp with the input optical power of 2.60 mW cm^−2^. In order to improve the performance of the cell, the system was irradiated using a Xe lamp (the input optical power of 43.7 mW cm^−2^) with 435 nm filter.

**1.4.3 Rotating Ring-Disk Electrode (RRDE) measurements**

RRDE measurements were performed in an O_2_-saturated sodium carbonate–bicarbonate buffer solution. The electrode was first activated by the cycling 15-20 times at a scan rate of 100 mV s^−1^, followed by CV measurements at a scan rate of 50 mV s^−1^. Linear sweep voltammetry (LSV) was operated with a scan rate of 5 mV s^−1^. The LSV curves were collected with different rotation rates of 900 rpm, 1250 rpm, 1600 rpm, 2025 rpm and 2500 rpm. The electron transfer number (*n*) and H_2_O_2_ production yield were determined from the following equation S1-2:^[7]^

$\text{n}\text{ = }\frac{\text{4 × }\text{I}_{\text{d}}}{\text{I}_{\text{d}}\text{ + }\frac{\text{I}_{\text{r}}}{\text{N}}}$	(S1)

$\text{H}\text{2}\text{O}\text{2}\text{ (\%) = }\frac{\text{2 × }\frac{\text{I}_{\text{r}}}{\text{N}}}{\text{I}_{\text{d}}\text{ + }\frac{\text{I}_{\text{r}}}{\text{N}}}\text{ × 100\%}$	(S2)

*I*_d_: the Disk Current Density of RRDE, mA·cm^−2^

*I*_r_: the ring Current Density of RRDE, mA·cm^−2^

*N*: the collection efficiency of the Pt ring, N = 0.37

**1.4.4 Solar-to-electricity energy conversion**

The solar-to-electricity energy conversion efficiency (*η*) is calculated according to equation S3-4:^[8]^

$\text{η}\text{ = }\frac{\text{P}_{\text{out}}}{\text{P}_{\text{in}}}\text{= }\frac{\text{V}_{\text{oc}}\text{ × }\text{I}_{\text{sc}}\text{ × }\text{FF}/{\text{A}_{\text{illuminated}}}}{\text{P}_{\text{hν}}}$	(S3)

$\text{FF}\text{ = }\text{P}_{\text{max}}\text{·}\text{I}_{\text{sc}}^{\text{ }-\text{1}}\text{·}\text{A}_{\text{illuminated}}\text{· }\text{V}_{\text{oc}}^{\text{ }-\text{1}}$	(S4)

Here, *P*_out_ is the electrical power output. *P*_in_ represents the photochemical energy input, and *P*_hν_ is the incoming photon energy. *A*_illuminated_ is the area of the electrodes exposed to the illumination. Here, it is the sum of *A*_photoanode_ and *A*_photocathode_. *V*_oc_ is the open-circuit voltage, *I*_sc_ is the short-circuit current, and *FF* is the fill factor. Note that *η* was calculated by using the maximum power density with the dark curve set as zero current. The solar-to-electricity conversion efficiency of the PEC cell under O_2_ and air atmospheres are shown in Table S3.

**1.4.5** **The measurement of Mott-Schottky (M-S) plots**

The Mott-Schottky (M-S) measurements were performed using a CHI 660E electrochemical workstation with a standard three-electrode system, in which the working electrode was the FTO plate the counter electrode was a graphite rod, the reference electrode was the Ag/AgCl (with saturated KCl aqueous solution) electrode, and the certain amount of N-CQDs(en) was dispersed in water with the identical conductivity and pH (Table S2) as the electrolyte. The flat-band potentials (*V*_fn_ and *V*_fp_) of p-n type N-CQDs(en) were determined using M-S plots at potentials varying from 0 V to 2.0 V (vs. RHE) with a frequency of 1 kHz. Based on the M-S equation shown in equation S5, a plot of $\text{1}/{\text{C}_{\text{SC}}^{\text{2}}}$ against *E* yields a straight line. The values of *V*_fn_ and *V*_fp_ were determined from the intercept on the E axis.^[9]^

$\frac{\text{1}}{\text{C}_{\text{SC}}^{\text{2}}}\text{ = }\text{−}\frac{\text{2}}{\text{ε}\text{ε}_{\text{0}}\text{E}\text{N}_{\text{d}}}\text{(}\text{E}\text{-}\text{E}_{\text{fb}}\text{-}\frac{\text{k}_{\text{B}}\text{T}}{\text{e}}\text{)}$	(S5)

Here, *C*_SC_ is the interfacial capacitance, *ε* is the dielectric constant of the semiconductor, *ε*_0_ is the permittivity of free space, *N*_d_ is the number of donors, *E* is the applied voltage, *E*_fb_ is the flat-band potential of semiconductor, *e* is the electronic charge, *k*_B_ is Boltzmann’s constant and *T* is the absolute temperature. The flat-band potential can be approximated as the Fermi level (*V*_f_) in the semiconductor ^[8].^ A positive slope indicates an n-type semiconductor, while a negative slope indicates a p-type semiconductor.

**1.4.6** **The calculation of the** **average charge decay lifetime in the Open circuit voltage decay (OCVD) test**

The calculation of the average charge decay lifetime was based on a reported method in the literature.^[10]^ The OCVD test was conducted to characterize the charge separation efficiency for N-CQDs(en) modified Cu_2_O photoelectrode, which gives a curve of photogenerated potential as a function of time. After light-off, the curve suddenly decays and reaches its value before the irradiation. A second-order nonlinear fitting is performed on the decay curve, and the average charge decay lifetime (*τ*_m_) of the system was calculated using the following formula (equation S6).^[10-11]^

$\text{τ}_{\text{m}}\text{= }\frac{\text{τ}_{\text{1}}\text{ × }\text{τ}_{\text{2}}}{\text{τ}_{\text{1}}\text{ + }\text{τ}_{\text{2}}}$	(S6)

Here, *τ*_1_ and *τ*_2_ is the fitting lifetime based on the second-order nonlinear fitting respectively.

**1. 5 Density functional theory (DFT) calculations**

DFT calculations were performed using Vienna *Ab initio* Simulation Package (VASP) ^[12]^. The interaction of core and valence electrons was treated with the projector augmented wave (PAW) pseudopotential,^[13]^ and the plane-wave cut-off energy was set to 520 eV. The exchange-correlation function was described using the generalized-gradient approximation-Perdew-Burke-Ernzerhof (GGA-PBE) method.^[14]^ The DFT-D3(BJ) method was used to account for the vdW-dispersion energy-correction.^[15]^ Geometry optimization was deemed converged when the force on each atom was less than 0.02 eV Å^−1^. Based on the experimentally identified predominant facet, a heterojunction model was constructed by coupling the Cu_2_O (111) surface with a carbon quantum dot model containing carboxyl groups, positively charged nitrogen, pyridinic nitrogen, and pyrrolic nitrogen. These models were employed to simulate the oxygen reduction reaction (ORR). The Brillouin zone was sampled using a Gamma-centered Monkhorst-Pack (MP) grids,^[16]^ and a k-point mesh of 3 × 3 × 2 was applied for structural optimization. Data analysis and visualization was facilitated by VASPKIT,^[17]^ QVASP^[18]^ and VESTA.^[19]^ The Gibbs free energy difference (ΔG) between initial and final states was denoted as formula (equation S7):

Δ*G* = Δ*E* + Δ*ZPE* -*T*Δ*S*	(S7)

where *E*, *ZPE*, *T* and *S* represent the energy from DFT calculation, zero-point energy, temperature (298.15 K) and entropy, respectively.^[20]^

**2. Tables and Figures**

**Figure S1.** The HR-TEM images of (a) N-CQDs(dmf) and (b) N-CQDs(am) respectively.

**Figure S2.** XRD spectra of a) N-CQDs(en), N-CQDs(dmf), N-CQDs(am) and b) Cu_2_O, respectively.

**Figure S3.** UV-vis spectra of aqueous solutions containing N-CQDs(en), N-CQDs(dmf), and N-CQDs(am), respectively.

**Figure S4.** Luminescence spectra of aqueous solutions containing a) N-CQDs(en), b) N-CQDs(dmf), and c) N-CQDs(am), respectively with different excitation wavelength.

**Figure S5.** XPS survey spectra and corresponding elemental compositions of the three N-CQDs: (a) N-CQDs(en), (b) N-CQDs(dmf), and (c) N-CQDs(am), respectively.

**Figure S6.** Zeta Potential of N-CQDs(en), N-CQDs(dmf), and N-CQDs(am).

**Figure S7.** LSV curves of N-CQDs(en), N-CQDs(dmf), N-CQDs(am), and Cu_2_O in the dark and under irradiation, in buffer solution using a three-electrode system. Note that at −0.10 V vs. Ag/AgCl electrode corresponding to 0.70 V vs. RHE, there is no electrochemical response for both Cu_2_O and N-CQDs in the dark.

**Figure S8.** a) The *J*–*V* curves and b) calculated power density plots of the PEC cells with N-CQDs(en) modified Cu_2_O photocathode with different electrodeposition times (0.5 h, 1 h, 1.5 h, 2 h respectively) under the irradiation using a 450 nm LED or the PEC cells with N-CQDs(en) modified Cu_2_O photocathode with 1.5 h electrodeposition under irradiation with a Xe lamp with 435 nm cutoff.

**Figure S9.** (a-b) The ORR LSV on different photocathodes (2 cm^2^) in a O_2_- or N_2_-saturated buffer solution, with or without irradiation using a 450 nm LED.

**Figure S10.** (a, c) The RRDE measurements, (b, d) the corresponding electron transfer numbers (*n*) and H_2_O_2_ production yields of the Cu_2_O and the N-CQDs(en), respectively in O_2_-saturated buffer solution.

**Figure S11.** The XPS spectra of a) C 1s, b) N 1s and c) O 1s for N-CQDs(en), Cu_2_O and N-CQD(en)/Cu_2_O photocathodes, respectively.

**Figure S12.** Tauc plot derived from the UV-vis spectrum of a N-CQDs(en) aqueous solution.

**Figure S13.** The LSV spectra of N-CQDs(en) in the a) anodic scan and b) cathodic scan, which was carried out in the corresponding N-CQDs(en) solution with a bare FTO electrode as working electrode, Ag/AgCl electrode as reference and graphite rod as a counter electrode.

The LSV curves afford the HOMO and LUMO of N-CQDs(en) at 1.92 eV and -0.16 eV respectively, giving an energy gap of 2.08 eV. This value agrees well with that obtained from optical absorption shown in Figure S10. In order to determine the band levels for both N-CQDs(en) and photoelectrodes, the HOMO or LUMO level of N-CQDs(en) is based on the potentials from the electrochemical measurements, and the other band location can be calculated in the combination of *E*_g_ obtained from optical absorption. Here, since the LUMO is located at -0.16 V for N-CQDs(en), it leads to HOMO at 1.87 eV based on 2.03 eV of energy band read from the UV-vis spectrum in Figure S10.^[21]^

**Figure S14.** The corresponding Tauc plot of Cu_2_O based on UV-vis DRS spectrum.

**Figure S15.** Mott-Schottky plots of the Cu_2_O photoanode in buffer solution.

Based on the UV-vis DRS spectrum in Figure S12, the corresponding Tauc plot of Cu_2_O provides an *E*_g_ of 2.01 eV. In the buffer solution, Cu_2_O shows a negative slope in a Mott-Schottky plot, consistent with the p-type semiconductor feature. Moreover, the intercept on the abscissa gives *V*_fb_ of Cu_2_O at 1.18 eV. Since the p-type semiconductor has a negligible gap between *V*_fb_ and the upper edge of the VB, the VB of Cu_2_O in the buffer solution is estimated as 1.18 eV, and the CB level as -0.83 eV.^[22]^

**Figure S16.** LSV on-off curves of the Cu_2_O and N-CQD(en)/Cu_2_O photocathodes in a buffer solution in three-electrode system under 450 nm LED illumination.

**Figure S17.** EIS plots of Cu_2_O and N-CQD(en)/Cu_2_O photocathodes in a three-electrode system under 450 nm LED illumination.

**Figure S18.** IPCE of the different photocathodes in buffer solution.

**Figure S19.** *I-t* curves of the Cu_2_O and N-CQD(en)/Cu_2_O photocathodes in buffer solution during the prolonged electrolysis under 450 nm LED illumination in a three-electrode system.

**Figure S20.** Charge density difference analysis of N-CQD(en)/Cu_2_O (The yellow and cyan bubbles represent electron accumulation and depletion regions, respectively; isovalue = 0.0005 e bohr^–3^).

**Figure S21.** **(**a) Electronic localization function (ELF) of N-CQD(en)/Cu_2_O. (b) The cross-sectional plot of the electron ELF for N‑CQD(en)/Cu_2_O.

**Figure S22.** (a) Charge density difference analysis of Cu_2_O. (b) Charge density difference analysis of O_2_-adsorbed N-CQDs(en) (isovalue = 0.005 e bohr^–3^).

**Figure S23.** LSV curves of a FeNiOOH/FeN-decorated BiVO_4_ photoanode under 450 nm LED irradiation and in dark conditions, respectively.

**Figure S24.** EIS plots of PEC cells with different photocathodes under 450 nm LED irradiation.

**Figure S25.** Effect of Gel/PCA-Na/N-CQDs(en) electrolyte thickness on the performance of the PEC cell with a FeNiOOH/FeN-decorated BiVO_4_ photoanode and a N-CQD(en)/Cu_2_O photocathode: a) The *J-V* curves, b) power density curves and c) EIS result, respectively.

**Figure S26.** Photographs of N-CQD(en)/Cu_2_O photocathodes in a standard PEC cell with different quasi-solid-state electrolytes before and after 15 h operation under 450 nm LED irradiation.

**Figure S27**. Cross-sectional SEM images of the N-CQD(en)/Cu_2_O photocathode (a) before and (b) after prolonged PEC operation.

Cross-sectional SEM measurements of the photocathode (N-CQD(en)/Cu_2_O) in the PEC electricity generation device with Gel/PCA-Na/N-CQDs(en) electrolytes were performed before and after prolonged PEC operation for 15 h. It indicates that the optimized PEC cell delivers the photocurrent retaining 90% of its initial value after 15 h of continuous operation (Figure 5d), and the SEM images reveal that the thickness of the N-CQD(en) layer on the electrode surface after long-term PEC operation displays no apparent change compared to the as-prepared electrode (Figure S27). This implies that the electrode modifier is continuously supplemented by fresh material. According to literature,^[23]^ the hydrophilic hydrogels can act as effective protective overlayers to immobilize cocatalysts in a 3D porous network for the enhancement of PEC activity and operational stability, These results together show that N-CQDs from the gel electrolyte are continuously replenished on the electrode surface, thereby ensuring the long-term stability and sustained operation of the electrode.

**Figure S28.** Photographs of the red LED light powered by two tandem-connected optimized cells, in which each comprises an N-CQD(en)/Cu_2_O photocathode and a FeNiOOH/FeN-decorated BiVO_4_ photoanode, with Gel/PCA-Na/N-CQDs(en) as the quasi-solid-state electrolyte, under the sustained illumination (commercial LEDs, λ = 450 nm with input optical power of 2.60 mW cm^−2^) for over 120 h.

**Figure S29.** Photographs of yellow and red LED lights powered by the PEC devices under the illumination of commercial LEDs (λ at 450 nm with input optical power of 2.60 mW cm^−2^).

**Figure S30.** Potential application scenario of the PEC cell as a light indicator in a green house.

**Table S1.** Electron transfer analysis of N-CQDs: positive/negative values represent electron gain/loss, respectively.

|  | Number of electrons transferred |
| --- | --- |
| Cu | –0.61 |
| O | 1.01 |
| O (Adsorbed oxygen) | 1.23 |
| N | 1.12 |
| C | –0.17 |
| H | –0.26 |

**Table S2.** The pH and conductivity of N-CQDs and sodium carbonate–bicarbonate buffer solutions.

|  | N-CQDs(en) | N-CQDs(dmf) | N-CQDs(am) | buffer |
| --- | --- | --- | --- | --- |
| pH | 9.99 | 10.08 | 10.15 | 10.05 |
| Conductivity (mS·cm^-1^) | 34.10 | 31.00 | 30.20 | 36.20 |

**Table S3**. Water retention capacity of Gel/PCA-Na and Gel/PCA-Na/N-CQDs after exposure to room conditions for 48 h.

| electrolytes | Water retention capacity (%) |
| --- | --- |
| Gel/PCA-Na | 69.70 |
| Gel/PCA-Na/N-CQDs(en) | 74.14 |
| Gel/PCA-Na/N-CQDs(dmf) | 68.74 |
| Gel/PCA-Na/N-CQDs(am) | 69.70 |

**Table S4**. The solar-to-electricity conversion efficiency of the gel-based PEC cell under O_2_ and air atmospheres.

| atmosphere | *V*_oc_ (V) | *I*_sc_  (mA) | *P*_max_  (mW) | *ŋ*  (%) |
| --- | --- | --- | --- | --- |
| air | 0.48 | 0.54 | 0.051 | 0.12 |
| O_2_ | 0.48 | 0.58 | 0.091 | 0.22 |

**Table S5**. Performance comparison of the PEC systems in this work with the reported unbiased dual-photoelectrode PEC cells.

| Irradiation source | Electrolyte | Electrode area (cm^2^) | Electricity output | Ref. |
| --- | --- | --- | --- | --- |
| λ= 450.0 nm LED lamp (2.6 mW cm^−2^) | electrolyte: Gel/PCA-Na/N-CQDs(en), pH = 9.99 | 4 | *V*_oc_: 0.48 V, *P*_max_:  0.091 mW | This work |
| AM 1.5G (100.0 mW cm^−2^) | 1 M NaOH,  pH = 14.0 | Photoanode: 1.8 cm^2^, photocathode: 1.6 cm^2^ | *V*_oc_: 0.90 V, *P*_max_:  0.22 mW·cm^−2^ | [8] |
| λ= 450.0 nm LED lamp (2.6 mW cm^−2^) | Photoanode electrolyte: N-CQDs, pH = 8.0; photocathode electrolyte: pH = 8.0 | 6 | *V*_oc_: 0.43 V, *P*_max_:  0.21 mW | [24] |
| AM 1.5G  light (100.0 mW cm^−2^) | Photoanode electrolyte: 1 M NaHCO_3_, pH = 8.3;  photocathode electrolyte: 0.5 M Na_2_SO_4_, pH = 6.0 | 9 | / | [25] |
| AM 1.5G  light (100.0 mW cm^−2^) | Photoanode electrolyte: 0.1 M KOH, pH = 13.0;  photocathode electrolyte: 1 M potassium borate buffer, pH = 9.5 | 9 | / | [26] |
| AM 1.5G  light (100.0 mW cm^−2^) | Photoanode electrolyte: 1 M H_2_SO_4_, pH = 0;  photocathode electrolyte: 1 M KOH, pH = 14.0 | 4 | / | [27] |

**3. References**

[1]	J. Liu, D. Li, K. Zhang, M. Yang, H. Sun, B. Yang, *Small* **2018**, *14*, 1703919.

[2]	R. Miao, S. Zhang, J. Liu, Y. Fang, *Chem. Mater.* **2017**, *29*, 5957-5964.

[3]	C. Xia, J. Zhong, X. Han, S. Zhu, Y. Li, H. Liu, B. Yang, *Angew. Chem. Int. Ed.* **2024**, *63*, e202410519.

[4]	L. Lan, J. Ping, H. Li, C. Wang, G. Li, J. Song, Y. Ying, *Adv. Mater.* **2024**, *36*, 2401151.

[5]	J. Yang, C. Deng, Y. Lei, M. Duan, Y. Yang, X. Chen, S. Yang, J. Li, H. Sheng, W. Shi, C. Chen, J. Zhao, *Angew. Chem. Int. Ed.* **2025**, *64*, e202416340.

[6]	X. Chang, T. Wang, Z.-J. Zhao, P. Yang, J. Greeley, R. Mu, G. Zhang, Z. Gong, Z. Luo, J. Chen, Y. Cui, G.A. Ozin, J. Gong, *Angew. Chem. Int. Ed.* **2018**, *57*, 15415-15419.

[7]	X. Li, X. Jin, Y. Cao, J. Zhu, D. Duan, Q. Luo, B. Lv, Y. Xu, Y. Zheng, W. Zhang, R. Long, L. Zhang, R. Liao, R. Cao, *J. Am. Chem. Soc.* **2025**, *147*, 43199-43205.

[8]	B. Zhang, L. He, T. Yao, W. Fan, X. Zhang, S. Wen, J. Shi, C. Li, *ChemSusChem* **2019**, *12*, 1026-1032.

[9]	B. Geng, J. Hu, Y. Li, S. Feng, D. Pan, L. Feng, L. Shen, *Nat. Commun.* **2022**, *13*, 5735.

[10]	J. Jian, S. Wang, Q. Ye, F. Li, G. Su, W. Liu, C. Qu, F. Liu, C. Li, L. Jia, A.A. Novikov, V.A. Vinokurov, D. H. S. Harvey, D. Shchukin, D. Friedrich, R. van de Krol, H. Wang, *Adv. Mater.* **2022**, *34*, 2201140.

[11]	C. Zhang, C. Xie, Y. Gao, X. Tao, C. Ding, F. Fan, H.-L. Jiang, *Angew. Chem. Int. Ed.* **2022**, *61*, e202204108.

[12]	a) G. Kresse, J. Furthmüller, *Comput. Phys. Commun.* **1996**, *6*, 15-50; b) G. Kresse, J. Furthmüller, *Phys. Rev. B* **1996**, *54*, 11169-11186.

[13]	a) G. Kresse, D. Joubert, *Physical Review B* **1999**, *59*, 1758-1775; b) P. E. Blöchl, *Phys. Rev. B* **1994**, *50*, 17953-17979.

[14]	J. P. Perdew, K. Burke, M. Ernzerhof, *Phys. Rev. Lett.* **1996**, *77*, 3865-3868.

[15]	S. Grimme, S. Ehrlich, L. Goerigk, *J. Comput. Chem.* **2011**, *32*, 1456-1465.

[16]	D. J. Chadi, M. L. Cohen, *Phys. Rev. B* **1973**, *8*, 5747-5753.

[17]	V. Wang, N. Xu, J.-C. Liu, G. Tang, W.-T. Geng, *Comput. Phys. Commun.* **2021**, *267*, 108033.

[18]	W. Yi, G. Tang, X. Chen, B. Yang, X. Liu, *Comput. Phys. Commun.* **2020**, *257*, 107535.

[19]	K. Momma, F. Izumi, *J. Appl. Crystallogr.* **2011**, *44*, 1272-1276.

[20]	a) J. K. Nørskov, J. Rossmeisl, A. Logadottir, L. Lindqvist, J. R. Kitchin, T. Bligaard, H. Jónsson, *J. Phys. Chem. B.* **2004**, *108*, 17886-17892; b) J. Zhang, J. Ma, T.S. Choksi, D. Zhou, S. Han, Y.-F. Liao, H.B. Yang, D. Liu, Z. Zeng, W. Liu, X. Sun, T. Zhang, B. Liu, *J. Am. Chem. Soc.* **2022**, *144*, 2255-2263.

[21]	T.-F. Yeh, C.-Y. Teng, S.-J. Chen, H. Teng, *Adv. Mater.* **2014**, *26*, 3297-3303.

[22]	Z. Tian, Z. Wang, Z. Ma, J. Li, X. Wang, *Sci. China Mater.* **2024**, *67*, 1564-1573.

[23]	J. Tan, B. Kang, K. Kim, D. Kang, H. Lee, S. Ma, G. Jang, H. Lee, J. Moon, *Nat. Energy* **2022**, *7*, 537-547.

[24]	H.-M. Duan, C.-G. Li, L.-M. Mo, J.-S. Dang, X.-H. Jia, J.-C. Yu, Y.-H. Mei, A. Thapper, H.-Y. Wang, *Adv. Sci.* **2025**, *12*, e17204.

[25]	S. Shi, Y. Song, Y. Jiao, D. Jin, Z. Li, H. Xie, L. Gao, L. Sun, J. Hou, *Nano Lett.* **2024**, *24*, 6051-6060.

[26]	W. Fan, B. Zhang, X. Wang, W. Ma, D. Li, Z. Wang, M. Dupuis, J. Shi, S. Liao, C. Li, *Energy Environ. Sci.* **2020**, *13*, 238-245.

[27]	T. H. Jeon, B. Kim, C. Kim, C. Xia, H. Wang, P. J. J. Alvarez, W. Choi, *Energy Environ. Sci.* **2021**, *14*, 3110-3119.
